# Exploring the exercise dose to induce symptomatic and biomarker change in endometriosis: A protocol for a randomised controlled trial

**DOI:** 10.1016/j.mex.2026.104107

**Published:** 2026-08-19

**Authors:** Tegan E Hartmann, Danielle Girard, Kelly A Baker, Cathy Hayles, Timothy D Miller

**Affiliations:** a School of Allied Health, Exercise and Sport Science, Charles Sturt University, Bathurst, NSW, 2795, Australia; b Alliance for Research in Exercise, Nutrition and Activity, Allied Health and Human Performance, Adelaide University, Adelaide, South Australia, Australia; c Movement & Me Exercise Physiology, Leabrook, Adelaide, South Australia, Australia

**Keywords:** Women’s health, Physical activity, Pain, Wellbeing, Conservative management

## Abstract

Exercise may be an effective tool to improve symptoms and quality of life in those with endometriosis. However, the optimal dose to elicit such changes is unknown. This study will explore the critical threshold of exercise dose required to elicit symptomatic and biomarker changes in women with endometriosis, particularly for those who may be disadvantaged through spatial and aspatial factors.

Fifty women with clinically confirmed endometriosis will be randomly allocated into one of four groups: a passive control, active control (yoga), short duration walking and yoga, long duration walking and yoga.

Participants in the three active groups will complete 12 weeks of exercise.

Symptom severity (chronic) and biomarkers will be assessed pre-post intervention; adherence, and symptom impact (acute) will be assessed each week.

## Graphical abstract

**Figure:**
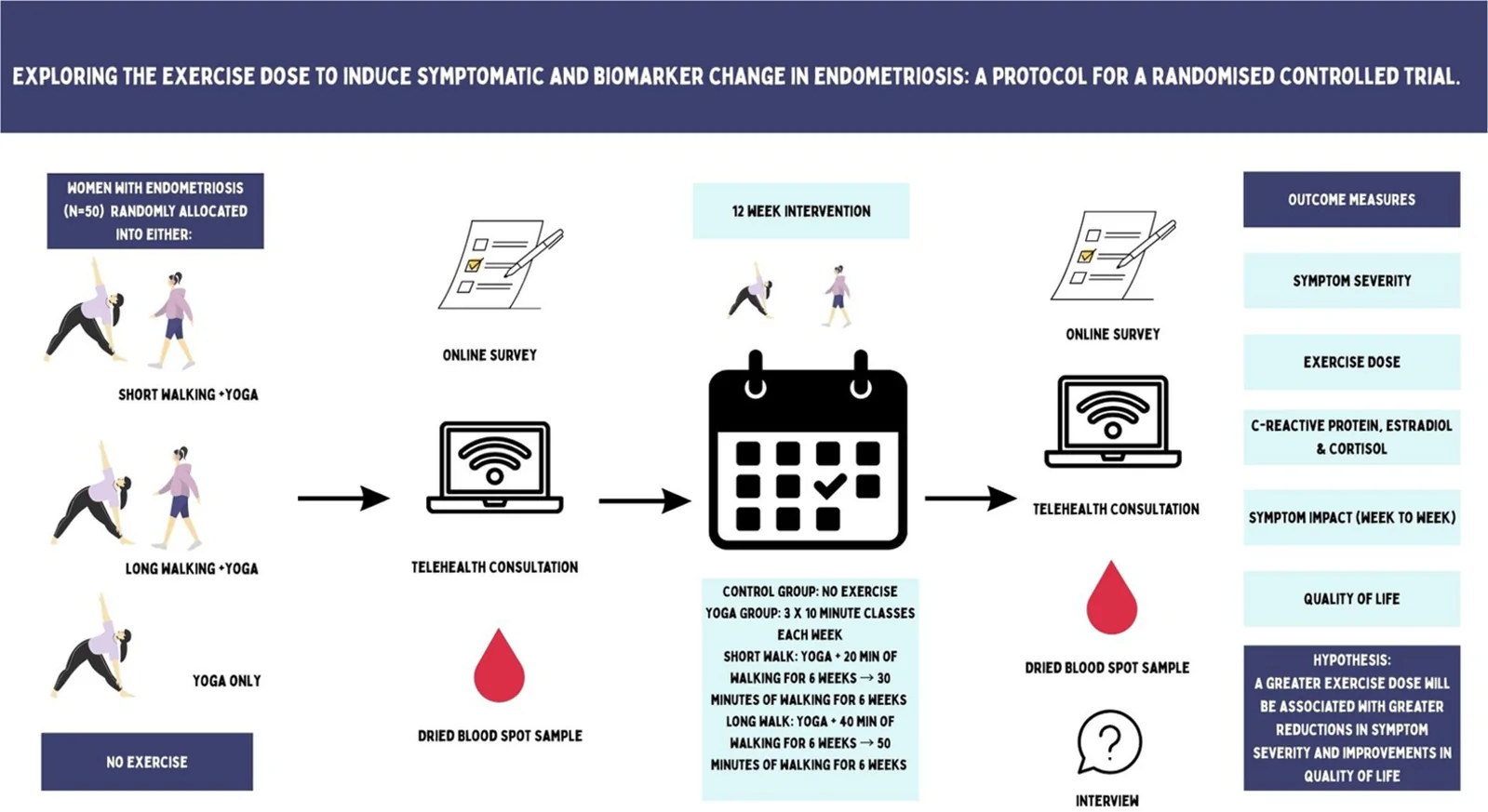

## Specifications table

**Table:** 

**Subject area**	Medicine and Dentistry
**More specific subject area**	Exercise Physiology
**Name of your protocol**	Exploring the exercise dose to induce symptomatic and biomarker change in endometriosis: A protocol for a randomised controlled trial.
**Reagents/tools**	Not applicable
**Experimental design**	This study will adopt a prospective, parallel randomized controlled trial design. Fifty women with clinically confirmed endometriosis will be randomly allocated into one of four groups: a passive control, active control (yoga), short duration walking and yoga, long duration walking and yoga. Participants in the three active groups will complete 12 weeks of exercise; symptom severity (chronic) and biomarkers will be assessed pre-post intervention; adherence, and symptom impact (acute) will be assessed each week. A combination of piecewise (segmented) mixed-effect regression, mixed model analysis and *t*-tests will be applied for analysis.
**Trial registration**	This protocol has been approved for registration on the Australian and New Zealand Clinical Trials Registry (ACTRN12625001442493p)
**Ethics**	Participants will be required to provide written and verbal consent following an outline of all procedures. This study conforms to the Declaration of Helsinki and Charles Sturt University’s Human Research Ethics (H25454) and Biosafety
**Value of the Protocol**	The protocol specifically itemizes the exercise prescription for women with endometriosis, allowing validation and enquiry in the field. The protocol is a self-directed home-based exercise intervention, which may promote adherence and longevity. The exercise prescription is low intensity with a low equipment requirement and can therefore assist in the servicing of disadvantaged populations. Both quantitative and qualitative data are collected, which will allow for a comprehensive, mixed methods assessment of the intervention both in terms of its effectiveness and feasibility / acceptability.

## Background

Recognized globally as a debilitating condition, endometriosis is a systemic disease involving endocrine, immune-inflammatory and proangiogenic processes[1]. While dysregulation in hormone-receptor signaling and immune-inflammatory pathways[2] are protagonists underlying symptom severity; neurogenesis, central sensitization, fibrosis, the location and size of endometriotic lesions, the organ systems affected, and the presence of comorbidities highlight the complexity of this condition. While the treatment of endometriosis is dictated by the chief complaint or primary indication[3], the most common forms of treatment are pharmacological and surgical. However, the reduction in symptoms is transient in nature; when medications are ceased, symptoms frequently return and further, post-operative recurrence is common[4]. Moreover, the economic burden associated with endometriosis can amount to $20 898 international dollars, per person, per year[5]. This impact is attributed not only to diagnosis and management, but also productivity losses[5,6]. When combined with spatial and aspatial factors that impact access to appropriate and timely treatment, such as geographical location, income and ethnicity[7], management of endometriosis becomes quite complex. Accordingly, further inquiry into potential alternative and sustainable management strategies is warranted.

Recent evidence suggests the management of endometriosis requires a multidisciplinary, life-course approach[8], extending beyond the reproductive system and incorporating physical and psychosocial elements, whilst acknowledging patient autonomy and self-management strategies[8].The importance of exercise and physical activity has gained increasing awareness as a viable non-pharmacological, non-surgical adjunct management strategy to support the quality of life of individuals with endometriosis[9]. Specifically, a recent meta-analysis reported physical activity and exercise to have beneficial effects on pain, quality of life, control and powerlessness and emotional well-being[10]. Exercise is widely recognized as an effective therapeutic intervention for pain management, fertility, cardiometabolic health, menstrual symptoms, indices of quality of life and improvement in inflammatory profiles[[11], [12], [13], [14], [15], [16]]. The potential of exercise to improve symptoms and quality of life in women living with endometriosis may be associated with immune and inflammatory modulation, regulation of autonomic function, musculoskeletal adaptations and reductions in central sensitisation[9]. However, globally, there are limited studies exploring specific exercise prescription (modality, volume, frequency and intensity) to improve symptoms, inflammation and quality of life. While emerging evidence suggests exercise has the capability to exert positive effects on symptoms, quality of life, biomarkers and even lesion size in endometriosis[[15], [16], [17], [18], [19], [20], [21], [22], [23]], to validate these findings, greater exploration into the exercise dose is required.

This study protocol has been designed to elucidate the critical threshold of exercise dose required to elicit changes in symptoms, quality of life, and biomarkers of endometriosis. By specifically detailing the exercise prescription, this protocol builds the evidence base for further scientific enquiry into exercise management in endometriosis. Further, this protocol provides clinical guidance to health and exercise professionals.

## Description of protocol

### Study design

This study will adopt a prospective, parallel randomized controlled trial design and aligns with the Standard Protocol Items: Recommendations for Interventional Trials (SPIRIT) checklist. Participants will be required to provide written and verbal consent prior to involvement. Once enrolled into the study, participants will complete an online survey to ascertain demographic information, current treatment, management strategies, fertility and loss, perceptions of exercise, current exercise behaviours and preferences, and scores of depression, stress, anxiety, pain, sleep quality, and quality of life. Participants will then complete an initial telehealth consultation which will involve the assessment of functional capacity variables. Furthermore, measures of CRP, estradiol, and cortisol will be analyzed via a dried blood spot sample.

Participants will be provided with an activity monitor (Garmin Vivosmart 5) for activity tracking and monitoring of adherence, and a dried blood spot collection kit. Symptoms and engagement in exercise will be tracked weekly and following the completion of the intervention. Following the completion of the intervention, participants will complete a final telehealth appointment with the research team for assessment of functional capacity variables. Following the completion of the intervention (6 weeks after), participants will be invited to participate in a short (approximately 20 min) one-on-one semi-structured interview to ascertain their lived experience during and following the intervention (Table 1).

**Table 1:** Participant timeline: Schedule of enrolment, interventions, and assessments.

Adapted from Chan et al. [24],.

### Patient and public involvement

The study intervention was informed by a consumer advisory group (CAG) conducted on the 14th of November 2025. The CAG included four women living with endometriosis (one providing written feedback in lieu of meeting attendance) and two accredited exercise physiologists who specialise in exercise management for endometriosis. The CAG provided feedback on the practicality of the intervention, the delivery of the intervention and provided symptom-based advice regarding the intensity and modality of the exercise prescription.

### Participants

Women (*n* *=* 50, assigned female at birth; AFAB; >18y) with medically diagnosed (imaging/ laparoscopy) endometriosis will be invited to participate. Recruitment will occur via various media outlets and will be ongoing in nature. Participants residing in the Australian Modified Monash Model (MMM) areas of MMM2-MMM7 will be invited to participate in the study. Participants must not be pregnant, breast-feeding or undergoing fertility treatment. Further, participants must not have had pelvic surgery, or an intrauterine device inserted or removed for the 6 months prior to beginning the study, and they must not have a diagnosis of adenomyosis. Moreover, participants who fail the 4-stage static balance test, are completing greater than 60 min of exercise per week or are currently using prescription steroids (prednisone or any derivative) will be excluded from the study.

### Pre-assessment and screening

Telehealth consultation (via Zoom) will include an overview of the study, instructions for blood spot sample, activity monitor, and the completion of basic functional capacity assessments. Participants will be screened for any medical condition that may exclude the individual from participating. Abdominal strength will be assessed via a plank duration hold, lower limb strength will be assessed via a wall sit endurance hold test, and global upper limb strength will be assessed by a handgrip dynamometer test, and balance will be assessed via a 4-stage balance test.

## Primary outcome measures

### Activity data

All participants will be provided with a Garmin Vivosmart5 activity monitor for remote activity tracking and adherence logging. Data regarding heart rate, exercise duration and modality will be collected weekly as part of the study. All participants, including passive controls, will be asked to document any incidental exercise completed outside of the study requirements using the Garmin Vivosmart5 activity monitor. The intensity of the walking and yoga sessions will be determined via obtaining the relative percentage of the average HR against age predicted MHR. Furthermore, internal training loads (TRIMP) will be calculated through: Training load = D x (Δ HR ratio) x 0.86e^1.67(Δ HR ratio)^. Moreover, MET-Minutes will be calculated as a secondary unit to measure exercise load.

### Survey data

Demographic information, current exercise behaviours (APSS), scores of depression, anxiety and stress (DASS-21), health-related quality of life (EHP-30), sleep (PSQI), self-efficacy (SEE) and open-ended questions regarding treatment, geographical location, fertility and access to care, will be collected pre- and post-intervention. Moreover, symptoms will be tracked weekly via the Endometriosis Impact Scale (EIS) through REDCap, a survey administration software program.

## Secondary outcome measures

### Biomarkers

Concentrations of estradiol, cortisol and CRP will be assessed via dried blood spot techniques and will be analyzed by Stratech Scientific (APAC, PTY, LTD), according to manufacturer’s instructions.

## Exercise intervention

This study will involve a remote 12- week exercise intervention for regional, rural and remote Australian women living with endometriosis. Participants will be randomly allocated into one of four groups: passive control, active control, shorter duration, longer duration. Allocation will be determined by simple randomization using a randomization table created by computer software (REDCap), with the sequence accessible to one member of the research team only (KB). Central randomization will be utilized for allocation. The passive control will involve usual care, and the active control group will complete 10 min of yoga, three times per week for the 12 weeks (supplementary file 1). The shorter and longer duration exercise groups will complete 10 min of yoga, three times per week in addition to a walking intervention. The shorter duration group will commence at 60 min per week (3 × 20-minute sessions), and the longer duration group will commence at 120 min per week (3 × 40-minute sessions). After six weeks, the total duration in each walking group will be increased to 90 min and 150 min, respectively, with each group increasing session duration by 10 min. Participants will be encouraged to walk as fast as comfortably possible for 1 min, every 5 min of each session.

## Semi-structured interview

Participants (including dropouts) will complete a one-on-one semi-structured interview with a member of the research team approximately six weeks after completing the 12-week exercise program. Interviews will be conducted via Zoom and will be approximately 20 – 30 min in duration. All interview data will be recorded and transcribed verbatim, before being analyzed via inductive thematic analysis. The purpose of the interviews is to gain an understanding of the participants’ experiences during and following the 12-week exercise intervention. The interviews will explore the unique barriers and facilitators to exercise adherence that women with endometriosis experience, and they will also provide an opportunity for participants to provide feedback about the exercise program that may inform changes to exercise prescription in future research work involving women living with endometriosis. Data obtained from the interviews will provide critical information about key characteristics that make women well suited to undertaking an exercise program whilst living with endometriosis. Depending on the results, an a posteriori triangulation of the qualitative and quantitative data may provide enhanced insight into the potential benefits of exercise for women living with endometriosis.

## Safety and monitoring

Participants will be monitored through their allocated Garmin device, with the option to log pain reports, if an exacerbation occurs. The chief investigator (TH) will be notified of the report, to initiate follow-up. Participants are encouraged to work within the limits of their symptoms and have no assigned day to complete the exercise, allowing for flexibility in participation. Moreover, the EIS questionnaire asks participants to reflect on their symptoms across the last 7 days, providing additional monitoring.

Participants are asked to notify the team in the event of an exclusion factor or something that may prohibit continued participation (for example, pregnancy or any condition/illness that makes it unsafe to proceed), and the research team will then discuss discontinuation with the trial and the exit process.

In the event of symptom exacerbation, two members of the research team (TH & TM) will meet with the participant and attempt to provide modifications that alleviate symptoms but ensure the fidelity of the initial underlying exercise prescription remains unchanged.

## Data analysis

All data will be de-identified for analysis and reporting. The estimated sample size was determined using data from Gonçalves et al.[25] where the mean difference for pain between the control and intervention was 24.74, for an effect size of d = 1.33 and comparisons between four groups, a sample size of 32 was estimated (alpha at 0.05 and power 0.80). Factoring in attrition and funding availability, the target sample size was set to 50. However, a piecewise (segmented) mixed-effect regression will be used to determine the critical threshold for exercise dose (primary outcome). Exercise dose (all randomized participants) will be determined by duration and intensity (heart rate) variables, aggregated weekly. For a sample of 50 participants across 12 weeks, with a mixed-effects piecewise model, there will be a minimum of 600 observations, which provide sufficient power and stability for this type of analysis[26]. Threshold and detectable change in slope will be determined by Y^t^ = B^0^ + B^1^* time^t^ + b^2^ * intervention^t^ + b^3^ time after intervention^t^ + e^t^, and confirmed with Davies Test. Intercurrent events such as medication changes, clinical exacerbations and poor adherence will be time- and date-stamped for consideration as co-variates in the regression analysis.

All other data will be reported as mean ± standard deviation (SD) or median interquartile ranges. Normal distribution will be determined by Shapiro-Wilk’s test and non-normally distributed data will be logarithmically transformed prior to analysis. Pain, as a subset of the EHP-30 is the primary endpoint and will be analysed with an intention-to-treat analysis. A sensitivity (per-protocol analysis) will also be conducted to confirm outcomes. Secondary and exploratory endpoints will be arranged hierarchically as follows; quality of life (EHP-30), depression, stress, anxiety (DASS-21), functional capacity, sleep (PSQI), estradiol, CRP, cortisol. Multiple comparisons will be adjusted for using the Benjamini-Hochberg false discovery rate procedure. Missing data will be managed depending upon the volume of data, complete case analysis will be performed (<5%) for 5–40%, multiple imputation for chained equations or random forest imputation will be employed. Complier average causal effect (CACE) analysis will be employed if compliance varies significantly. Significance will be set at p < 0.05. All statistical procedures will be performed using Statistical Package for the Social Sciences (SPSS; Windows version 31.0, Chicago, IL, USA) and R Studio (2026.07.1).

## Ethics and dissemination

Participants will be required to provide written and verbal consent following an outline of all procedures. This study conforms to the Declaration of Helsinki and has institutional ethics and biosafety approvals. Findings will be shared through publication and presentation at industry conferences and through collaborations with endometriosis advocacy groups.

## Limitations

This study is not without limitations. To address the spatial factors affecting endometriosis care, the mode of delivery is telehealth, as such this presents limitations with monitoring, adherence and attrition. As the exercise prescription is self-directed, adherence may be low, and attrition may result, and this may then reduce the power and observations. Moreover, the use of the activity monitor may vary depending upon user (logging physical activity), and therefore, some data may not be as reliable as laboratory-based data. Further, biomarker sampling, conducted via self-collected dried blood spots, was random and therefore not aligned with the menstrual cycle. Accordingly, data may not be indicative of maximal inflammatory and hormonal load associated with the condition and should be interpreted with caution. Finally, this study upheld strict exclusion criteria, so findings may not be applicable to individuals who present with comorbidities such as adenomyosis. To counter these limitations, future studies should consider fully supervised and individualized interventions.

## Related research article

None.

## CRediT authorship contribution statement

**Tegan E Hartmann:** Conceptualization, Methodology, Project administration, Funding acquisition, Resources, Validation, Writing – original draft, Writing – review & editing. **Danielle Girard:** Conceptualization, Methodology, Validation, Writing – review & editing. **Kelly A Baker:** Methodology, Validation, Writing – review & editing. **Cathy Hayles:** Methodology, Validation, Writing – review & editing. **Timothy D Miller:** Conceptualization, Methodology, Validation, Writing – review & editing.

## Declaration of competing interest

The authors declare that they have no known competing financial interests or personal relationships that could have appeared to influence the work reported in this paper.

## Data Availability

No data was used for the research described in the article.

## Appendix Supplementary materials

Supplementary material *and/or* additional information [OPTIONAL]. Supplementary Table 1- The Yoga Intervention. This supplementary file details the specifics of the yoga intervention, for replicability purposes, which in previous bodies of work has been omitted. The publication of this protocol is a step forward in reporting research and transparency of interventions. Image, application 1
