# Supplementary material for: Exploring the exercise dose to induce symptomatic and biomarker change in endometriosis: A protocol for a randomised controlled trial
Source: MethodsX. 2026 Aug 19;17:104107. doi: 10.1016/j.mex.2026.104107 (PMC13542919; doi:10.1016/j.mex.2026.104107)
Supplement: Supplementary file 1 [file mmc1.docx]

Supplementary File 1: Yoga Intervention

| Class | Sequence | Notes |
| --- | --- | --- |
| 1 | Childs pose with side stretch (balasana, side stretch variation), cat-cow (marjaryasana–bitilasana), downward facing dog (adho mukha svanasana), low lunge (anjaneyasana), half splits (ardha hanumanasana), twisted dragon (parivrtta utthan pristhasana), gate pose (parighasana), reclined butterfly (supta baddha konasana), supine twist (supta matsyendrasana), corpse pose (savasana). | **Each class was delivered in the sequence it has been presented here.**  Duration: 20 minutes.  Variations offered: In twisted dragon and reclined butterfly- using support to ensure control in the movements.  As the first class, extra instructions were provided. |
| 2 | Reclined butterfly (supta baddha konasana), windscreen wiper legs  (supine spinal twist flow), half happy baby  (ardha ananda balasana),  reclined hamstring stretch  (supta padangusthasana)  supine twist (supta matsyendrasana), knees to chest (apanasana), easy pose (sukhasana), downward facing dog (adho mukha svanasana)  low lunge (anjaneyasana)  garland pose (malasana)  pigeon pose (kapotasana)  corpse pose (savasana). | Duration: Approximately 10 minutes.  Variations offered: In garland pose- lessen the depth, if causing too much pelvic strain. |
| 3 | easy pose with side stretch (parsva sukhasana), butterfly pose (baddha konasana), seated hamstring stretch  (ardha baddha konasana, variation forward bend), gate pose (parighasana), cat-cow  (marjaryasana–bitilasana), downward facing dog, (adho mukha svanasana), low side extend (utthita parsvakonasana), gentle twist (parivrtta utthita ashwa sanchalanasana), low lunge (anjaneyasana), half splits (ardha hanumanasana), bridge pose (setu bandhasana), corpse pose (savasana). | Duration: Approximately 10 minutes. |
| 4 | child’s pose (balasana),  cat-cow (marjaryasana–bitilasana), gate to extended child’s pose (adductor rock) (parighasana, flow variation), puppy pose  (uttana shishosana),  oscillating cobra (bhujangasana, flow variation), half frog (ardha bhekasana), downward facing dog (adho mukha svanasana), three- legged down dog (tri pada adho mukha svanasana), low lunge with prayer twist  (parivrtta anjaneyasana), low warrior 2 (virabhadrasana ii variation eka janu bhumi), happy baby (ananda balasana), reclined pigeon with drop to either side (supta kapotasana), corpse pose (savasana). | Duration: Approximately 10 minutes.  Variations offered: Leaving the foot of the non-stretched leg on the floor during reclined pigeon. |
| 5 | child’s pose (balasana), camel to moving camel (ustrasana, flow variation), downward facing dog (adho mukha svanasana), sun salutation a (surya namaskar a), goddess pose (utkata konasana)  wide forward fold (prasarita padottanasana), knees to chest (apanasana), supine twist  (supta matsyendrasana), cat-tail pose (supine spinal twist variation), corpse pose (savasana). | Duration: Approximately 10 minutes.  Variations offered: Options for regression with depth of goddess squat. |
| 6 | corpse pose (savasana)  knees to chest, (apanasana), windscreen wiper legs (supine spinal twist flow), wide knees to chest (apanasana, wide variation), cat-cow  (marjaryasana–bitilasana),  downward facing dog  (adho mukha svanasana), garland pose, (malasana), gentle twist (parivrtta utthita ashwa sanchalanasana), high lunge (ashta chandrasana), warrior 2  (virabhadrasana ii), triangle pose (trikonasana), mountain pose (tadasana)  bridge pose (setu bandhasana), reclined half frog (vishraam ardha bhekasana), reclined butterfly (supta baddha konasana). | Duration: Approximately 10 minutes.  Variation offered: Low (on knees) variation provided as option. |
| 7 | crocodile pose (makarasana), sphinx pose (alamba bhujangasana),  quadriceps stretch (ardha bhekasana variation),  downward facing dog (adho mukha svanasana),  low lunge (anjaneyasana), low twisted reverse warrior (viparita parivrtta anjaneyasana),  low reverse warrior (kneeling viparita virabhadrasana), seated twist (ardha matsyendrasana), pigeon  (kapotasana), rock baby (hindolasana), corpse pose (savasana) | Duration: Approximately 10 minutes. |
| 8 | easy pose (sukhasana), deer pose (mrigasana), cat-cow (marjaryasana–bitilasana), downward facing dog (adho mukha svanasana), garland pose  (malasana), garland pose -rise to circle (malasana-flow variation), high lunge  (ashta chandrasana) humble warrior (baddha virabhadrasana), low lunge (anjaneyasana)  half splits (ardha hanumanasana),  bridge pose (setu bandhasana), happy baby  (ananda balasana), cat-tail pose (supine spinal twist variation), corpse pose (savasana) | Duration: Approximately 10 minutes. |
| 9 | child’s pose with side stretch (balasana-side stretch variation),  cat-cow (marjaryasana–bitilasana),  bound camel (ustrasana -variation), low side plank (vasisthasana), camel pose (ustrasana), low lunge (anjaneyasana), twisted dragon  (parivrtta utthan pristhasana), low lizard  (utthan pristhasana)  high lunge (ashta chandrasana), forward fold (uttanasana), knees to chest drop either side (apanasana -side drop variation), half happy baby  (ardha ananda balasana),  supine twist (supta matsyendrasana), corpse pose (savasana). | Duration: Approximately 10 minutes. |
| 10 | corpse pose (savasana)  knees to chest (apanasana), easy pose with side stretch (parsva sukhasana), downward facing dog (adho mukha svanasana), garland pose  (malasana), gentle twist with cactus arm (parivrtta utthita ashwa sanchalanasana),  high lunge (ashta chandrasana), goddess pose (utkata konasana)  side lunge (skandasana),  bridge pose (setu bandhasana), butterfly pose (baddha konasana)  corpse pose (savasana). | Duration: Approximately 10 minutes.  Variations offered: Having the feet closer together, and therefore limiting depth, during side lunge. |
| 11 | Child’s pose (balasana), rolling cat-cow (marjaryasana–bitilasana , flow variation) low lunge (anjaneyasana), half splits (ardha hanumanasana), low side extend (utthita parsvakonasana), camel pose (ustrasana), butterfly with forklift arms (baddha konasana shoulder stretch variation), sphinx pose  (alamba bhujangasana), quadriceps stretch (ardha bhekasana variation), reclined pigeon (supta kapotasana), corpse pose  (savasana). | Duration: Approximately 10 minutes. |
| 12 | Crocodile pose (makarasana), cobra  (bhujanga), half frog pose (ardha bhekasana),  half bow pose (ardha dhanurasana), cat-cow (marjaryasana–bitilasana), low lunge (anjaneyasana), low side plank (vasisthasana), gate pose (parighasana), high lunge (ashta chandrasana),  pyramid pose (parsvottanasana),  downward facing dog  (adho mukha svanasana),  stork pose (saaras pakshi asana), cross forward fold (uttanasana with cross),  pigeon (kapotasana),  supine twist (supta matsyendrasana),  downward facing dog (adho mukha svanasana),  corpse pose (savasana). | Duration: Approximately 10 minutes.  Variations offered: Reaching toward foot, rather than holding the foot, in half bow pose. |
